# Transmastoid Ultrasound Detection of Middle Ear Effusion and Its Association with Clinical Audiometric Tests

**DOI:** 10.3390/life12040599

**Published:** 2022-04-18

**Authors:** Chin-Kuo Chen, Yung-Liang Wan, Li-Chun Hsieh, Po-Hsiang Tsui

**Affiliations:** 1 Department of Otolaryngology-Head and Neck Surgery and Communication Enhancement Center, Chang Gung Memorial Hospital, Taoyuan 333423, Taiwan; dr.chenck@gmail.com; 2 School of Traditional Chinese Medicine, College of Medicine, Chang Gung University, Taoyuan 333323, Taiwan; 3 Department of Otolaryngology-Head and Neck Surgery, Chang Gung Memorial Hospital, Keelung 204201, Taiwan; 4 Department of Medical Imaging and Intervention, Chang Gung Memorial Hospital at Linkou, Taoyuan 333423, Taiwan; ylw0518@cgmh.org.tw; 5 Department of Otolaryngology-Head and Neck Surgery, Mackay Memorial Hospital, Taipei 104217, Taiwan; 6 Department of Audiology and Speech Language Pathology, Mackay Medical College, Taipei 104217, Taiwan; 7 Department of Medical Imaging and Radiological Sciences, College of Medicine, Chang Gung University, Taoyuan 333323, Taiwan; 8 Division of Pediatric Gastroenterology, Department of Pediatrics, Chang Gung Memorial Hospital at Linkou, Taoyuan 333423, Taiwan

**Keywords:** otitis media with effusion, tympanometry, ultrasound

## Abstract

Medical history taking, otoscopy, tympanometry, and audiometry are clinical methods to diagnose middle ear effusion (MEE); however, these procedures are experience-dependent and result in misdiagnosis under unfavorable conditions of the external auditory canal in non-cooperative young children. This study aimed to explore the use of transmastoid ultrasound combined with the Nakagami parameter analysis to detect MEE in children aged 3–5 years and to compare the proposed method with clinical evaluation methods. A total of forty subjects were enrolled; for each subject, a single-element ultrasound transducer of 2.25 MHz was used to measure backscattered signals returned from the mastoid for estimating the Nakagami parameter, which is a measure of the echo amplitude distribution. Tympanogram and hearing loss were also measured for comparisons. The results showed that the Nakagami parameter in the patients with MEE was significantly larger than that of the normal group (*p* < 0.05). The area under the receiver operating characteristic curve (AUROC) for using the Nakagami parameter to detect MEE was 0.90, and the sensitivity, specificity, and accuracy were 82.5%, 97.5%, and 79.6%, respectively. The Nakagami parameter for tympanogram types B/C was higher than that for tympanogram type A (*p* < 0.05); it was also higher in the subjects with hearing loss (*p* < 0.05). Quantitative transmastoid ultrasound based on the Nakagami parameter analysis has the potential to detect MEE and evaluate hearing loss.

## 1. Introduction

Acute otitis media (AOM) usually presents with otalgia, restless sleep, and hearing impairment, and it is the second most common cause for children to visit the pediatrician [1]. AOM is characterized by the presence of acute inflammation with middle ear effusion (MEE). It is estimated that 80% of all children will suffer from otitis media in their lifetime, and most of them will experience otitis media with an effusion before they reach school age [2]. Persistent MEE may result in conductive hearing loss [3,4], resulting in a 20–30 decibel (dB) hearing loss, affecting speech ability and the quality of life and leading to learning delay [4], especially in children aged from 2 to 5 years old. Myringotomy and needle tympanocentesis with fluid aspiration remain the gold standard methods for diagnosing MEE. However, they are difficult to perform in young children because of their invasiveness and the requirement of anesthesia.

According to the clinical practice guidelines, pneumatic otoscopy is strongly recommended for diagnosing MEE in children. For young children, a relatively narrow ear canal and impacted cerumen may interfere with the otoscope’s ability to inspect the condition of the tympanic membrane, leading to misdiagnosis. Tympanometry is another method recommended to assist in the diagnosis of MEE, which usually presents as type B (middle ear involvement from fluid, a perforation, or patent grommet) or C (eustachian tube dysfunction, which is often seen just before or after effusion). When a child has MEE, audiometry is an important tool for assessing hearing sensitivity. The average hearing loss in children with MEE is 27 dB [5]. MEE that causes more than 30 dB of average pure-tone hearing loss may be less likely to resolve spontaneously [6], and these cases are at risk of learning problems and delay in language development, and a possible ventilation tube insertion surgery is recommended. However, audiometry and tympanometry require cooperation between audiologists and children. The need for air-tight sealing of the ear canal makes it challenging to achieve an accurate diagnosis in an uncooperative young child. Recently, an optical coherence tomography (OCT) otoscope has gradually received attention and emerged as a new tool for MEE characterization. By using a low-intensity light source, OCT produces real-time structural images for tissue differentiation and detection at micron scales. OCT otoscopy proved to be promising for the accurate detection of MEE [7,8]. Nonetheless, the imageability of the OCT otoscope was affected by age; younger children were difficult to image, while older children were relatively easy to image, similar to current ear diagnostics [7]. Thus, it would be helpful and appealing to develop an alternative and noninvasive method to overcome the limitations in measuring children.

The mastoid cavity is an air-filled space in a bony structure, which is connected to the middle ear cavity and is influenced by MEE. It has been confirmed that mastoid effusion (ME) influences the intensity of ultrasound signals backscattered from the microstructures when ultrasound is transmitted into the mastoid [9]. Our previous study [10] further proposed a strategy based on using an ultrasound transducer to be placed on the surface of the mastoid to measure MEE-induced ME by estimating the Nakagami parameter to quantify changes in the statistical properties of signals. Notably, according to the clinical practice guidelines published by the American Academy of Otolaryngology–Head and Neck Surgery, clinicians should offer tympanostomy tube insertion to children with bilateral otitis media effusion or definitive diagnosis of over 3 months and document hearing difficulties [11]. Considering some of the exudates in the middle ear cavity may disappear after AOM for 6 weeks, we need a point-of-care tool to support routinely quantitative examination and follow-up in addition to using otoscopy or tympanometry. In this condition, quantitative transmastoid ultrasound based on the Nakagami parameter analysis is a potential modality for detecting MEE in children, but it has not been investigated and applied to patients aged 3–5 years. The dependencies of the Nakagami parameter of the mastoid on MEE history, tympanogram type, and hearing loss are also unanswered.

The aim of this study is to explore the diagnostic performance of using the Nakagami parameter in detecting MEE in patients aged 3–5 years. In the Materials and Methods section, we explain how we enrolled subjects, conducted measurement procedures, and analyzed data. The Results section reports the Nakagami parameters of the mastoid for comparisons with the findings of MEE history, tympanogram type, and hearing loss for discussion. The Discussion section compares the results with some existing works, interprets underlying mechanism of change in the Nakagami parameter, and addresses the significance and limitations of this study. The Conclusions section indicates the main contribution of this study; that is, transmastoid ultrasound using Nakagami parameter analysis can detect MEE in children as well as distinguish between tympanogram types and subjects with and without hearing loss, offering a solution for routine examinations and follow-ups to children with potential AOM.

## 2. Materials and Methods

### 2.1. Subject Enrolment

This study was approved by the Joint Institutional Review Board of Chang Gung Memorial Hospital (No. 201600985B0). Children were presented to our outpatient department with ear discomfort or suspected otitis media as reported by their families from January 2018 to December 2018. A total of forty participants aged 3–5 years were enrolled in this study. Informed consent was obtained from each patient and their relatives.

### 2.2. Clinical Assessments

A total of 20 healthy volunteers without any medical histories in MEE-related problems participated in the normal group (40 ears), and another 20 subjects that had been diagnosed as having bilateral MEE and scheduled for being undergoing grommet surgery to drain effusion were enrolled in the MEE group (40 ears). All subjects were re-examined through otoscopy by an experienced otolaryngologist; after that, their ear canals were sealed using a 226 Hz probe tone for tympanometry measurements by professional audiologists. Subsequently, audiologic evaluations by pure tone audiometry (PTA) were performed. Interacting with children who are undergoing clinical examinations can be very challenging, and thus conditioned play audiometry was used to improve cooperation. Air conduction and bone conduction hearing levels at 0.5, 1, 2, and 4 kHz were recorded because the speech spectrum typically falls into the range between 500 to 4000 Hz. Children in the normal group were confirmed to have tympanogram type A and normal otoscopic findings; those with the confirmed diagnosis of MEE were identified following otoscopic findings in accompanying with tympanogram type B/C and surgical findings fed back from the surgical team. The sample size was determined by the software G*power [12] (version 3.1, Heinrich-Heine-University, Düsseldorf, Germany), indicating that the sample size in each group should be at least 8 to achieve a power of 0.95 in a test based on α = 0.05.

### 2.3. Ultrasound Measurement

Transmastoid ultrasound examination of each subject was performed using an ultrasound system and the algorithm proposed in a previous report [10]. The system consisted of a portable pulser-receiver (Model USB-UT350, US Ultratek, Inc., Martinez, CA, USA), a 2.25-MHz delay-line single-element transducer (V204-RM, Panametrics-NDT, Waltham, MA, USA), and a personal computer. The purpose of using a delay-line transducer is to separate the excitation pulse and the echoes backscattered from the mastoid by using a short piece of plastic or epoxy material attached in front of the transducer surface. The transducer was positioned on the mastoid with coupling gel between the mastoid and transducer to allow acoustic wave propagation. The pulser-receiver with a built-in 60 dB amplifier and analog-to-digital converter (the sampling rate: 25 MHz) was used to drive the transducer transmitting ultrasound into the mastoid space for acquiring backscattered signals, which were stored in the personal computer for off-line data processing. For each backscattered signal, its absolute value of the Hilbert transform was calculated to obtain the corresponding envelope signal for estimating the Nakagami parameter of the Nakagami distribution by (Equation (1))

(1) $$m=\frac{{\left[E\left(R^{2}\right)\right]}^{2}}{E{\left[R-E\left(R^{2}\right)\right]}^{2}}$$

where *E*(·) is the statistical mean operator, and *R* is the envelope signal. The probability density function *f*(*r*) of the Nakagami distribution for the envelope signal *r* is given by (Equation (2))

(2) $$f\left(x\right)=\frac{2m^{m}x^{2m-1}}{\Gamma \left(m\right)\Omega^{m}}exp(-\frac{m}{\Omega }x^{2})U\left(x\right)$$

where *Γ*(·) and *U*(·) are the gamma function and the unit step function, respectively. The scaling parameter *Ω* is related to echo energy. The Nakagami parameter is a shape parameter of the Nakagami distribution, and it is able to describe various scattering conditions in the tissue. Values of the Nakagami parameter <1 means that the echo amplitude distribution is the pre-Rayleigh distribution, while a value = 1 indicates the Rayleigh distribution for the backscattered statistics. In this condition, higher Nakagami parameter values represent that the scattering medium belongs to a more supportive environment for producing echoes. Five independent measurements were carried out to obtain an average Nakagami parameter for each subject.

### 2.4. Statistical Analyses

Typical backscattered signals measured from the normal control and the MEE groups were plotted. The Nakagami parameters of the two groups were expressed as median and interquartile range (IQR) and compared using the independent *t*-test; *p* value < 0.05 was considered a statistically significant difference. To evaluate the diagnostic value of the Nakagami parameter in determining the presence of MEE, the receiver operating characteristic (ROC) curve analysis at 95% confidence interval (CI) was performed to obtain the area under the ROC (AUROC). The sensitivity, specificity, and accuracy were also reported. Nonparametric tests were used for small sample size data. Additionally, the data in the normal control and MEE groups were further regrouped to explore the effects of MEE duration, tympanogram type, and hearing loss on the Nakagami parameter of the mastoid. All statistical analyses were performed using the Statistical Package for the Social Sciences (SPSS) version 22.0 (SPSS, Inc., Chicago, IL, USA, IBM Company, New York, NY, USA).

## 3. Results

### 3.1. Patients’ Characteristics

Twenty children without MEE (14 boys and 6 girls, 4.2 ± 1.0 years old) as the normal group and 20 children with MEE (12 boys and 8 girls, 4.4 ± 1.1 years old) as the MEE group were enrolled. There were no significant differences in the age and sex between the groups (*p* = 0.65 and *p* = 0.74, respectively).

### 3.2. Ultrasonic Signals in Both Groups

**Figure 1:**
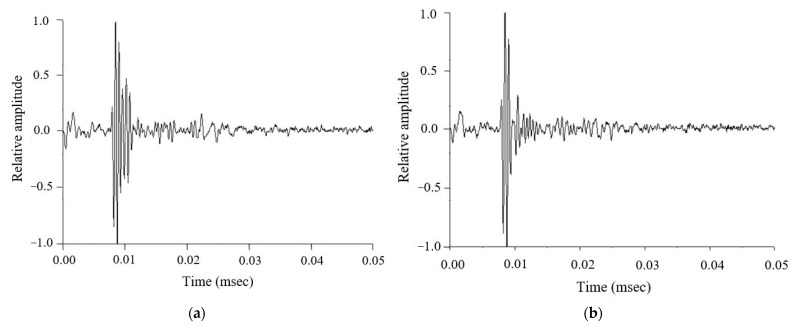
Typical ultrasound backscattered signals measured from the mastoid. (**a**) Normal group; (**b**) MEE group.

Figure 1 shows the ultrasound backscattered signals of the mastoid obtained from the normal control and MEE groups, respectively. The signals between 0 and 0.01 ms are background signals corresponding to the delay-line material of the transducer, and those approximately located at 0.01 ms are the reflection signals returned from the mastoid surface. Ultrasound backscattered signals received after 0.01 ms were contributed by microstructures (i.e., air cells) in the mastoid. The backscattered signals tended to be large and exhibited a relatively high variation in amplitude for the normal control; those obtained in the MEE group tended to have a lower degree of variance in amplitude. The difference in the waveform between the two groups may be attributed to effusion-induced changes in the acoustic impedance in the mastoid.

### 3.3. Nakagami Parameters in the Normal and MEE Groups

**Figure 2:**
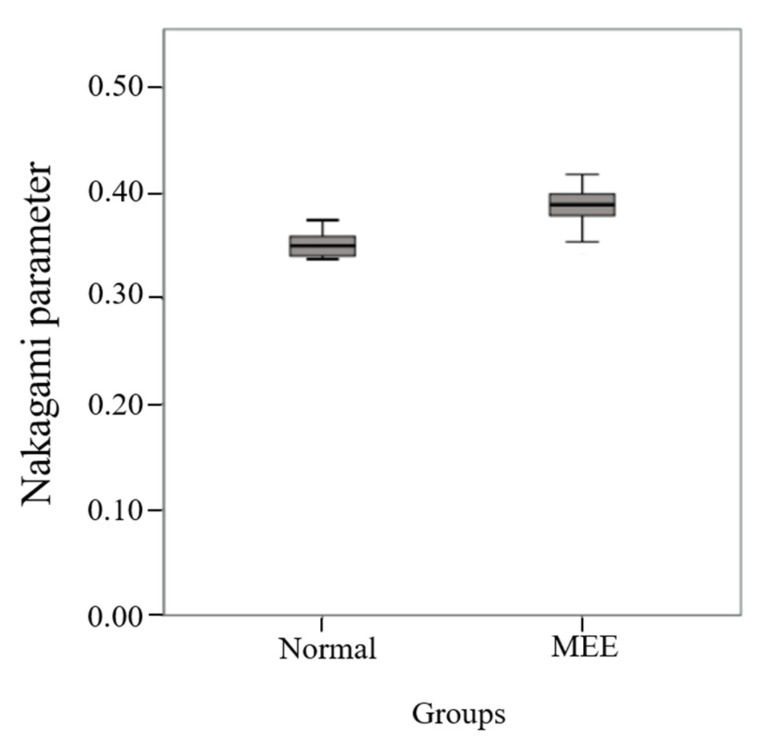
The Nakagami parameters obtained from the normal and MEE groups. The Nakagami parameter for the MEE group was higher than that of the normal control (*p* < 0.001).

**Figure 3:**
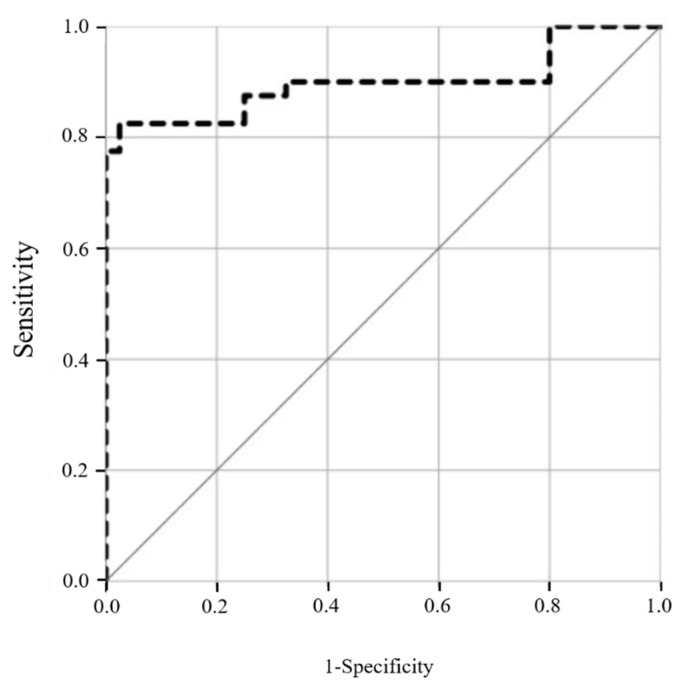
The ROC curve for using the Nakagami parameter to diagnose MEE. The AUROC was 0.90 (95% CI: 0.82–0.98), and the sensitivity, specificity, and accuracy were 82.5%, 97.5%, and 79.6%, respectively.

The statistical properties of ultrasound backscattered signals were further quantified by estimating the Nakagami parameter. The median of the Nakagami parameter for the MEE group was 0.39 (IQR: 0.38–0.40), which was larger than 0.35 (IQR: 0.34–0.36) for the normal group, as shown in Figure 2. There was a significant difference in the Nakagami parameter between the groups (*p* < 0.001), indicating that the echo amplitude distribution of the mastoid tends to vary toward the Rayleigh statistics when the middle ear is filled with effusion. As shown in Figure 3, the AUROC for using the Nakagami parameter to diagnose MEE was 0.90 (95% CI: 0.82–0.98), and the sensitivity, specificity, and accuracy were 82.5%, 97.5%, and 79.6%, respectively.

### 3.4. Nakagami Parameters in MEE within a Period of 3 Months and Longer than 3 Months

**Figure 4:**
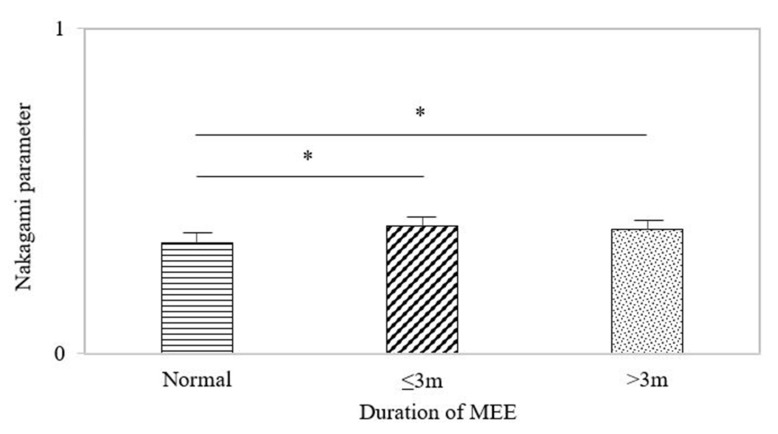
Nakagami parameter data classified according to different MEE durations. No significant difference existed between MEE ≤3 months and MEE >3 months. Significant differences were found between the normal and MEE ≤3 months and between the normal and MEE >3 months. The symbol ‘*’ means *p* < 0.05.

The data of the Nakagami parameter in the normal control and MEE groups were further classified by the MEE duration of <3 months and >3 months, as shown in Figure 4. The Nakagami parameters with MEE history ≤3 months and >3 months were 0.39 ± 0.03 and 0.38 ± 0.02, respectively, indicating no significant difference. However, significant differences were found between the normal and MEE ≤3 months (*p* < 0.05) and between the normal and MEE >3 months (*p* < 0.05).

### 3.5. Nakagami Parameters in the Three Types of Tympanogram

**Figure 5:**
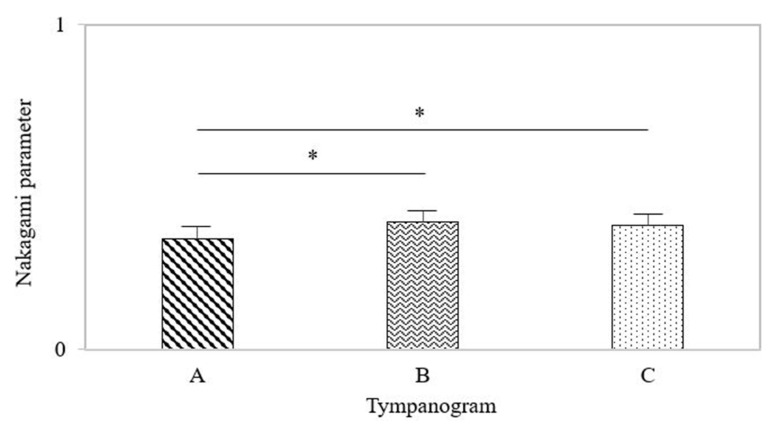
The Nakagami parameters for tympanogram types A, B, and C. No significant difference existed between the types B and C. Significant differences in the Nakagami parameter were found between the types A and B and between the types A and C. The symbol ‘*’ means *p* < 0.05.

The Nakagami parameters corresponding to different types of tympanograms were compared, as shown in Figure 5. The Nakagami parameters for the types A, B, and C were 0.34 ± 0.04, 0.39 ± 0.03, and 0.38 ± 0.03, respectively. No significant difference existed between the types B and C. In comparison, significant differences in the Nakagami parameter were found between the types A and B (*p* < 0.05) and between the types A and C (*p* < 0.05). 

### 3.6. Nakagami Parameters in the MEE Group with Hearing Loss ≤30 dB and >30 dB

**Figure 6:**
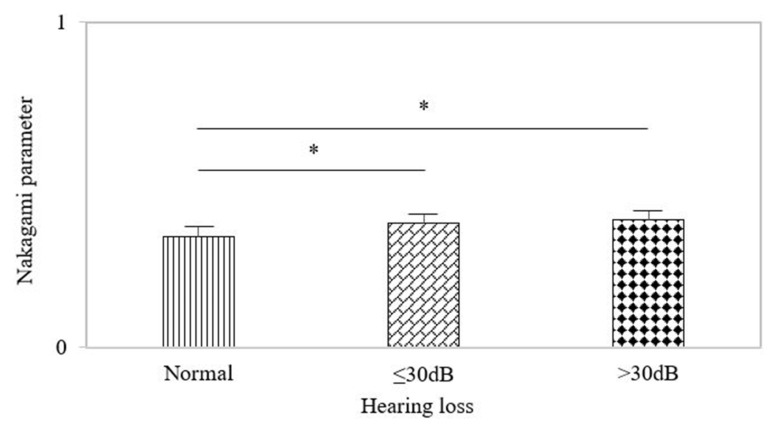
The Nakagami parameters in the MEE group with hearing loss. The Nakagami parameter was able to discriminate between the normal control and the subjects with hearing loss. However, it was unable to stage the level of hearing loss. The symbol ‘*’ means *p* < 0.05.

The data of the Nakagami parameter in the MEE group were compared according to the levels of hearing loss ≤30 dB and >30dB, as shown in Figure 6. The Nakagami parameters for hearing loss ≤30 dB and >30dB were 0.38 ± 0.02 and 0.39 ± 0.03, respectively; the above Nakagami values were significantly higher than that of the normal control (*p* < 0.05). However, the Nakagami parameter was independent of the level of hearing loss although it was able to discriminate between the normal control and the subjects with hearing loss.

## 4. Discussion

Standard diagnostic tools of MEE, such as pneumatic otoscopy, which is considered a visual assessment method, are often unreliable if patients are uncooperative. The diagnostic accuracy of traditional otoscopy for MEE detection varies between 40 and 70% [13]. The diagnostic sensitivity and specificity of the pneumatic otoscope are 94% and 80%, respectively [14]. The diagnostic sensitivity, specificity, and accuracy of tympanometry are 85.9%, 72.2%, and 83.8%, respectively [15]. Machine learning algorithms have also been applied to otoscopy for automated effusion detection, offering accuracy of 90.3%, sensitivity of 90.5%, and specificity of 90.1%; however, the amount of data used for training influences the diagnostic performance, and the depth of otoscope insertion affects the light intensity in regions of interest, making it difficult to capture the entire extent of the tympanic membrane [16]. In this study, quantitative transmastoid ultrasound was investigated, and the Nakagami parameter estimated using ultrasound backscattered signals measured from the mastoid was found to detect MEE with diagnostic sensitivity of 82.5%, specificity of 97.5%, and accuracy of 79.6%. Transmastoid ultrasound based on the Nakagami parameter analysis is comparable to current clinical diagnostic tools in MEE detection, but has not been ideal compared with machine learning-assisted otoscopy, although the proposed method belongs to a physical rule-based methodology and does not need a large amount of data for training and testing prior to its applications in practice.

Besides MEE detection, we also explored the dependencies of the Nakagami parameter on MEE history, tympanogram type, and hearing loss. Patients with otitis media for >3 months are at risk of hearing loss [17], which is an indication for tympanostomy tube insertion in children. The current findings indicated that the patients with both ≤ and >3 months of MEE history showed very close results in the Nakagami parameter. This indicates that the Nakagami parameter of the mastoid was unable to predict the duration of MEE, which may be due to the lack of sensitivity in tissue characterization. In general, increasing ultrasound frequency may improve the sensitivity of using ultrasound statistical parameters to characterize microstructures [18]. Nevertheless, using higher frequencies sacrifices penetration depth of acoustic waves, especially for bone tissues due to the acoustics attenuation effect. According to the comparisons presented in previous studies [9,10] and clinical experience, the ultrasound transducer with the central frequency of 2.25 MHz used in this study is still recommended for practical measurements on the mastoid in order to have a tradeoff between sensitivity and the penetration depth.

Considering another risk factor for ventilation tube insertion, hearing loss >30 dB for the speech frequencies (300 to 3000 Hz) is unacceptable for conversational communication [19] because of the reduction in the ability in language and speech learning. Although no significant difference in the Nakagami parameter was found between hearing loss ≤30 dB and >30 dB, the Nakagami parameter had the ability to discriminate between the normal subjects and those with hearing loss. The predisposing factors that influence hearing loss include the presence of MEE, the condition of the tympanic membrane, and ossicular chain mobility, implying that the Nakagami parameter not only reflects MEE but also depends on the properties of the tympanic membrane and ossicular chain. It should be noted that the Nakagami parameters corresponding to tympanogram types B and C were larger than that for tympanogram type A. Tympanogram types B and C mean more effusion in middle ear cavity compared to tympanogram type A. This may explain why the Nakagami parameter increases in the condition of MEE. When fluid fills with air cells in the mastoid, it causes constructive wave interference to strengthen ultrasound backscattering in the microstructures, making the echo amplitude distribution vary in the direction of the Rayleigh distribution to increases the Nakagami parameter [10]. The proposed method and device may be beneficial as a complement to conventional MEE detection methods, improving decision making regarding grommet insertion and possibly decreasing unnecessary surgeries and the administration of disease-resistant antibiotics in the future. For example, according to the latest report [20], many children with AOM or MEE who were originally scheduled for tympanostomy tube placement were unable to undergo surgery due to COVID-19 quarantine policies; interestingly, those children experienced resolution of their AOM or OME after a period of quarantine. In this instance, implementation of point-of-care measurement systems integrated with quantitative transmastoid ultrasound could enable in-home routine screening to not only prevent unnecessary medical treatments but also reveal risks for subjects who have not undergone surgery.

This study has some limitations. First, the number of subjects is insufficient, and the middle ear cavity individually varies; thus, a large-scale, randomized control study is necessary to explore considerations in practical uses prior to using quantitative transmastoid ultrasound as a reliable tool to evaluate MEE in a clinical setting. Second, there are multiple factors, including volume, fluid viscosity, mucosal thickness, E tube dysfunction, and bacterial toxins, that influence the hearing of patients. The dependencies of the Nakagami parameter on the above factors should be further clarified to specifically confirm the role and position of quantitative transmastoid ultrasound in MEE detection. Third, children aged under 3 years were not enrolled in the study. More pediatric cases need to be included and validated. Finally, the statistical properties of ultrasound backscattered data may differ between different systems. Developing a system dedicated to transmastoid ultrasound measurement and an optimized computational algorithm is a key future work.

## 5. Conclusions

We have explored the diagnostic performance of using quantitative transmastoid ultrasound based on the Nakagami parameter to detect MEE in children aged 3–5 years. The AUROC was 0.90, and the sensitivity, specificity, and accuracy were 82.5%, 97.5%, and 79.6%, respectively. It demonstrated that the underlying mechanism that MEE produces mastoid effusion to further affect the statistical properties of ultrasound backscattered signals measured from bony microstructures can be applicable to children, endowing the Nakagami parameter of the mastoid with the ability to detect pediatric MEE. In addition, the Nakagami parameter of the mastoid also had the ability to discriminate between tympanogram types A and B/C and between the subjects without and with hearing loss, but it was unable to identify how long the MEE history had been due to the lack of sensitivity in tissue characterization with low-frequency ultrasound. In the future, transmastoid ultrasound combined with the Nakagami parameter can be used for detecting MEE and evaluating hearing loss, providing routine examinations and follow-ups to children with potential AOM.

## Data Availability

The data presented in this study are available on request from the corresponding author.
